# Advanced adenoid cystic carcinoma of the cervix: a case report and review of the literature

**DOI:** 10.4076/1757-1626-2-6634

**Published:** 2009-06-16

**Authors:** Lalla Kawtar Elhassani, Hind Mrabti, Nabil Ismaili, Youssef Bensouda, Ouafae Masbah, Imane Bekkouch, Khalid Hassouni, Fouad Kettani, Hassan Errihani

**Affiliations:** 1 Department of Medical Oncology, National Institute of OncologyAgdal/Riad, Rabat-10000Morocco; 2 Department of Radiotherapy, National Institute of OncologyAgdal/Riad, Rabat-10000Morocco; 3 Department of Pathology, Ibnsina HospitalAgdal/Riad, Rabat-10000Morocco

## Abstract

**Introduction:**

Adenoid cystic carcinoma is a malignant epithelial neoplasm derived from the salivary glands. Primary adenoid cystic carcinoma of the cervix is extremely rare, accounting for less than 1% of all cervical carcinomas. In this paper we report a case of primary adenoid cystic carcinoma and a review of the related literature.

**Case presentation:**

A 68 year-old woman was admitted with signs and symptoms suggestive of a cervical cancer. The radiological and pathological investigations confirmed the diagnostic of primary adenoid cystic carcinoma of the cervix at Stage IIIB according to the International Federation of Gynaecology and Obstetrics classification. The patient was managed successfully by concurrent chemo-radiotherapy.

**Conclusion:**

The optimal management of adenoid cystic carcinoma cannot be established for certain. From our case and from the literature, it appears that combined treatment (surgery, radiotherapy, and chemotherapy) is necessary for achieving a long-term remission. Concurrent chemo-radiotherapy appears to be a logical option for locally advanced disease.

## Introduction

Adenoid cystic carcinoma (ACC) is a malignant epithelial neoplasm derived from the salivary glands and can occur in a variety of other sites. It is characterized by slow growth and high rate of local recurrence. Metastatic spread is usually a long-term complication [1]. Primary ACC of the cervix is extremely rare, accounting for less than 1% of all cervical carcinomas [1]. The first case was described in 1949 by Paalman [2]. To our knowledge, only 160 cases have been reported up to 2008. Because of the rarity of the disease, no standard treatment has yet been proposed. We report a case of primary ACC of the cervix and we discuss briefly the clinical and therapeutic features of the disease.

## Case presentation

**Figure 1.**
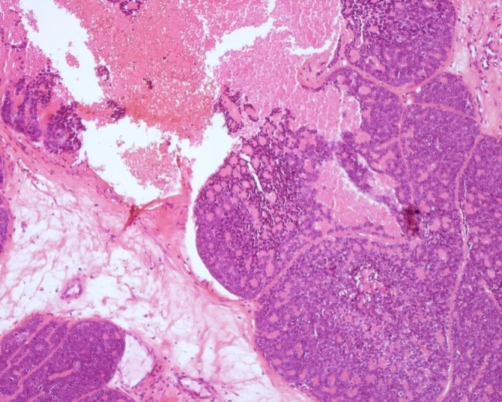
Hematoxylin and eosin (HE) staining of the biopsy specimen, low power view: Proliferation composed of small, basaloid cells with trabecular structure. Cylindromatous structures were present. The cribriform pattern was barely seen with large areas of necrosis.

**Figure 2.**
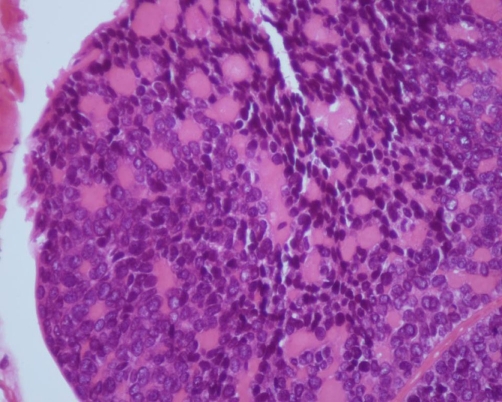
Hematoxylin and eosin (HE) staining of the biopsy specimen, high power view.

A 68 year-old postmenopausal Caucasian woman (para 10, gravida 10) admitted to our hospital with spontaneous vaginal bleeding for 6 months. The patient had never used *oral* contraceptives or hormone replacements. Gynaecological examination showed an exophytic ulcerated tumor measuring up to 8 cm in the largest diameter. The lower third of the vagina was involved by the tumoral process. On bimanual recto-vaginal examination we showed that the tumour had invaded both sides of the parametrium. Pelvic computed tomography (CT) scan showed a heavily cervical mass measuring 6 cm × 10 cm. The cervical biopsy was performed. Histological study of the sampled material showed adenoid cystic carcinoma infiltration (Figures 1 and 2). CT scan of the chest and abdomen was normal. The patient was staged IIIB according to the FIGO classification (classification systems established by the International Federation of Gynaecology and Obstetrics for the staging of gynaecological cancers). She was managed with concurrent chemotherapy and photon (25MV) external-beam radiotherapy (RT). The total dose of RT was 70 Gy delivered in 2 Gy daily fractions with concurrent weekly cisplatin chemotherapy at a dose of 40 mg/m^2^ iv for 6 cycles. After the treatment completed, the symptoms disappeared with complete response of the tumour in physical examination and in CT scan. The patient, 12 months after the end of chemoradiotherapy, remains disease free. She is continuously followed by our group up to now.

## Discussion

We report a case of locally advanced ACC of the cervix a rare malignancy in general. Primary ACC of the cervix accounts for less than 1% of all cervical carcinomas [1]. The origin of this disease is still unknown. Although, Human papillomavirus (HPV) infection is believed to be a necessary cause of cervical cancer, its role in the pathogenesis of ACC is not well defined [3]. There are some controversies concerning the epidemiological features of this tumor. Our patient was post-menopausal, Caucasian and multiparous woman. ACC of cervix was considered as the disease of the postmenopausal women [4], but more recent data reported some cases in young woman [5-8]. Although, some authors suggested the association between ACC, high parity and black race [4,9], Dixit supported the absence of the observed relationship [10].

Clinical and radiological characteristics of primary ACC of the cervix were similar to those of a squamous cell cancer. The main symptom of disease was the vaginal bleeding [10]. In the review reported by Dix et al, the most majority of patients were diagnosed at early stages, while only 10 cases were diagnosed at stage IIIB and 4 at stage IV [10].

Because of the rarity of the disease and the absence of prospective studies, no standard treatment has yet been proposed. Most patients were treated as squamous cell carcinoma. Surgery seems to be the treatment of choice in combination with adjuvant radiotherapy and/or chemotherapy, based on the clinical stage and presence of metastasis. Dixit recommended surgery with adjuvant CRT for early stage, and CRT in advanced stage [10]. A large number of chemotherapeutic regimens have been used in disease management. The most used chemotherapeutic drug is CDDP given at dose 100 mg/m^2^. Other regimens have been investigated, such as bleomycin, adriamycin and 5 Fluoro-uracil. To our knowledge, our patient is the 13th Stage IIIB of ACC and the first case successfully managed with concurrent CRT. (Table 1) summarizes the clinical findings of majority of published Stage IIIB ACC of the cervix.

**Table 1.** Clinical findings of majority of published stage IIIB ACC of the cervix

Author	Year	Age	Treatment	Follow-up	Status
Gallager [9]	1971	64	RT	30 months	DOD
Fowler [4]	1978	72	RT	6 years	DOD
Hoskins [6]	1979	40	RT	2 months	DOD
Prempree [11]	1980	52	RT	-	DOD
Miles [12]	1981	-	RT	9 months	DOD
Shingleton [13]	1981	78	RT	2,5 months	DOD
Musa [14]	1985	-	RT	11 month	NED
Berchuck [15]	1985	79	RT+CT	-	Progressive disease
Berchuck [15]	1985	72	RT	7 months	Peritoneal metastasis
Dixit [5]	1994	30	RT	48 months	NED
Nishida [16]	2005	78	RT	5 years	NED

Abbreviations: RT: Radiotherapy; CT: Chemotherapy; DOD: Death of disease; NED: No evidence of disease.

ACC of the cervix seems to be more aggressive than squamous cell carcinoma of the cervix, with higher tendency to local and metastatic recurrence even if diagnosed in their earliest stages [1,17]. Five years and ten years survival were 37% and 40% respectively [18]. These tumours spread most frequently to the lung, lymph nodes, abdominal cavity and brain [10]. The prognostic factors identified were: large tumour diameter, deep stromal invasion, and presence of tumor cells within a lymphatic space or capillary/ blood [19]. Consequently, the authors support the use of multimodality therapy including surgery, chemotherapy and radiotherapy for achieving long term remission [10].

## Conclusion

In the absence of prospective study, optimal management for ACC of the cervix cannot be established for certain. From the literature, it appears that combined treatment is necessary for achieving cure. Radical surgery with adjuvant radiotherapy and/or chemotherapy appears to be a logical option. Concurrent chemo-radiotherapy should also be considered for locally advanced disease.

## Abbreviations

ACC: Adenoid cystic carcinoma
CDDP: Cisplatin
CT: Computed tomography
CRT: Chemo-radiotherapy
FIGO: International Federation of Gynaecology and Obstetrics
HPV: Human papillomavirus
MV: Megavolt
RT: Radiotherapy
